# Prevalence of common autosomal recessive and X-linked conditions in pregnant women in Vietnam: a cross-sectional study

**DOI:** 10.1038/s41598-025-03399-5

**Published:** 2025-05-30

**Authors:** Trang Thi Nguyen, Ha Thu Thi To, Anh Ngoc Thi Le, Anh Quang Pham, Nhu Duc Nguyen, Hao Huu Ha, Huyen Thi Vu, Thanh Thai Hoang, Minh Cong Tran

**Affiliations:** 1 Hanoi Medical University, Hanoi, Vietnam; 2 Hanoi Medical University Hospital, Hanoi, Vietnam; 3 ClinGen Asia Testing Clinic, Hanoi, Vietnam; 4 University of Medicine and Pharmacy, Hanoi, Vietnam; 5 VinUniversity, Hanoi, Vietnam; 6 Thai Binh University of Medicine and Pharmacy, Thai Binh, Vietnam; 7 National Institute of Forensic Medicine, Hanoi, Vietnam; 8 Institute of DNA Technology and Genetic Analysis, Hanoi, Vietnam; 9 Nuffield Department of Clinical Neuroscience, University of Oxford, Oxford, England, UK

**Keywords:** Autosomal recessive screening, X-linked recessive screening, Recessive carrier screening, Vietnamese pregnant women, G6PD, Genetics, Cancer genomics, Clinical genetics

## Abstract

The prevalence of recessive disorder carriers among Vietnamese women is still indistinct. This study aims to assess the prevalence of carriers for common autosomal recessive and X-linked conditions among Vietnamese pregnant women and to identify common mutations within these genes. A cross-sectional study was conducted with 8,464 Vietnamese pregnant women with indications for carrier screening tests for recessive disorders from November 2022 to August 2023 at the Institute of DNA Technology and Genetic Analysis. The survey includes demographic information, and the genetic screening was conducted using next-generation sequencing (NGS) techniques, focusing on 13 specific recessive conditions. 8,464 Vietnamese pregnant women’s records were involved in this study. 1,928 of them carried at least one genetic recessive condition, representing the frequency of a recessive disorder was 22.8%. The highest recessive disorders rate among pregnant women was found for the G6PD gene mutation (G6PD deficiency) at a rate of about 1 in 20 individuals, followed by the HBA1 and HBA2 gene mutations (Alpha Thalassemia) at a rate of about 1 in 25. Other common recessive carrier genes included SRD5A2 (5-alpha reductase deficiency) at a rate of about 1 in 27, HBB (Beta Thalassemia) at a rate of about 1 in 28, ATP7B (Wilson’s disease) at a rate of about 1 in 40, PAH (Phenylketonuria) at a rate of about 1 in 40, and SLC25A13 (Citrin deficiency) at a rate of about 1 in 45. The prevalence of recessive carriers among Vietnamese pregnant women is high, and at least 1 in 5 pregnant women carries one recessive gene. It is essential to encourage Vietnamese pregnant women to conduct recessive carrier screening tests to reduce mortality rates among children and to implement effective pregnancy planning and childbirth.

## Introduction

Autosomal recessive (AR) and X-linked conditions affect approximately 1 in 300 pregnancies globally^1^. These days, over 2500 identified types of recessive genetic disorders, displaying an incidence of approximately 0.053 per 1000 live children for autosomal recessive conditions and 1.84 per 1000 live children for X-linked conditions^2,3^. These conditions can lead to result in intrauterine death, neonatal death, or chronic disability, contributing to approximately 20% of infant mortality and 10% of infant hospitalizations worldwide^4^. Due to their hereditary nature at the gene level, most recessive genetic conditions are currently incurable. Treatment for individuals with these conditions constantly involves specialized therapies such as special diets (e.g., phenylketonuria), enzyme therapy (e.g., Pompe’s disease), regular blood transfusions, and iron elimination (e.g., thalassaemia) during their lifespan. Individuals with recessive genetic conditions face a high risk of premature death, with mortality rates ranging from 80 to 90% without appropriate care, but with optimal treatment, it can reduce this rate to 28%^5,6^. Therefore, early diagnosis and screening of recessive genetic conditions are crucial to prevent severe complications, reduce treatment costs, and alleviate the financial strain on pediatric patients, their families, and society^7–9^. Since many carriers of AR and X-linked conditions show no symptoms, carrier screening is essential for couples to assess their risk of having a child with a recessive condition and consider alternative reproductive choices. Hence, the American College of Medical Genetics and Genomics (ACMG) established a connection between the effectiveness of carrier screening and reproductive decision-making in 2013^10^. With the advanced technologies in genetic testing, ACMG provided an overlapping tiered approach to carrier screening, which includes four tiers: (Tier 1) Cystic fibrosis + Spinal muscular atrophy + Risk-Based Screening; (Tier 2) ≥ 1/100 carrier frequency (includes Tier 1); (Tier 3) ≥ 1/200 carrier frequency (includes Tier 2) and includes X-linked conditions; and (Tier 4) < 1/200 carrier frequency (includes Tier 3) genes/condition will vary by lab. On top of that, Tier 2 and Tier 3 screenings prioritise conditions based on their carrier frequency within the general population, and Tier 3 carrier screening should be offered to all pregnant patients, especially^2^. However, even though following the guideline for selecting conditions for a panel gene of carrier screening for pregnant women was developed by the American College of Obstetricians and Gynecologists (ACOG), the lack of clarity in these recommendations can lead to varying interpretations among carrier screening providers, resulting in significant differences in the screening panels offered on the market^11^. Furthermore, recent advancements in next-generation sequencing (NGS) have enabled the simultaneous screening of numerous disorders, regardless of ethnicity, within a single test. This can reduce the price and costs for preconception and prenatal screening to expand the population using this test. Hence, unlike in the past, when carrier screening was primarily accessible to high-risk individuals, the more affordable pricing now allows for more comprehensive access to this valuable service.

According to a recent study by Tran Ngoc Hieu et al., nine recessive disorders were identified as the most common carrier genes among the Vietnamese population, including G6PD deficiency, Phenylketonuria, Citrin deficiency, Galactosemia, 5 alpha-reductase deficiency, Pompe disease, Wilson disease, Alpha Thalassemia, and Beta Thalassemia^12^. Moreover, Cystic fibrosis is recommended by ACMG to all people around the world because of its high prevalence and the level of severe Cystic fibrosis disease. Due to Vietnamese genetic experts’ recommendations for carrier screening testing, some diseases are required to be screened in Vietnam such as Glutaric acidemia type 2, Fabry disease, and Tay-Sachs disease. Therefore, in this study, utilizing a panel carrier screening with 13 recessive conditions, which was adopted by ACMG and Vietnamese experts’ recommendations regarding Vietnam backgrounds and ethnicities to identify the prevalence of these carriers and explore the common mutations of the most recessive conditions among Vietnamese pregnant women.

In Vietnam, significant disparities in healthcare access across regions have posed challenges to understanding the prevalence of recessive genetic diseases. The diversity in healthcare service distribution policies between different regions, particularly between urban centers like Hanoi and Ho Chi Minh City and rural areas, has created significant barriers to conducting large-scale studies to survey and estimate the proportion of people carrying recessive genetic diseases. Urban areas benefit from advanced healthcare infrastructure and greater access to genetic screening services, while rural and remote regions struggle with limited resources, insufficient medical personnel, and lower awareness of genetic conditions, leading to uneven participation rates and data collection difficulties that hinder a complete national assessment of carrier status. This leads to an incomplete list of recessive genetic diseases with high incidence. This is the first study to provide a comprehensive overview of genes associated with recessive genetic conditions screened among pregnant women at the national level. It also simultaneous identification of variants of the most common recessive genes based on the largest Vietnam database. Moreover, the results of this study will be guidelines for the provision of genetic counselling to reduce the incidence of recessive genetic conditions in the fetus and aid in more effective pregnancy planning and childbirth.

## Method

### Study methods and population

The data were collected from 8,464 Vietnamese pregnant women, predominantly Vietnamese Kinh ethnicity, from urban and semi-urban Northern Vietnam, representing diverse ethnicities and socioeconomic statuses, all Vietnamese-speaking. They underwent recessive carrier screening tests at the Institute of DNA Technology and Genetic Analysis in Hanoi, Vietnam. This cross-sectional study spanned 10 months, from November 2022 to August 2023, and was conducted at the Institute of DNA Technology and Genetic Analysis.

Selection criteria: The study enrolled asymptomatic pregnant women who voluntarily chose carrier screening as part of their prenatal care, irrespective of any documented familial history of genetic disorders. Participants were required to have an indication for non-invasive prenatal screening, with the focus on identifying carrier status for recessive conditions, despite none exhibiting symptoms of the screened disorders.

Exclusion criteria: Participants were excluded from the study if they declined to provide informed consent, had insufficient or compromised sample quality, or did not meet the inclusion criteria of being pregnant and opting for carrier screening. These criteria were established to ensure that only consenting individuals with viable samples and appropriate eligibility were included in the research. This has been detailed in the “Methods” section of the study.

### Implementation

A sample of 7–10 ml of peripheral venous blood was collected from each participant and preserved in an EDTA tube. Samples were collected following strict laboratory guidelines, with quality control measures implemented at each stage. These samples were then analyzed using next-generation sequencing processed by the Illumina NextSeq platform at the Institute of DNA Technology and Genetic Analysis to identify 13 recessive gene conditions. The detected variants were aligned to the GRCh37 human reference genome, and variant calling was performed using GATK. They were annotated using databases such as NCBI, Varsome, and OMIM for pathogenicity assessment. For this study, a carrier frequency cut-off of 1.7% (1 in 60) was applied to classify common disorders based on the genetic landscape of recessive diseases in the Vietnamese population reported by Tran et al.^12^. Variants of recessive disorders with a prevalence exceeding 1 in 60 was added to the Vietnamese-specific carrier screening panel.

### Panel design for 13 recessive genes

As the screening of recessive carrier genes is still unfamiliar in Vietnam and the standard panel gene is not identified for all Vietnamese women, we used a panel carrier screening with 13 autosomal recessive and X-linked conditions, including G6PD deficiency, Phenylketonuria, Citrin deficiency, Galactosemia, 5 alpha-reductase deficiency, Pompe disease, Wilson disease, Alpha Thalassemia, and Beta Thalassemia, Cystic fibrosis, Glutaric acidemia type 2, Fabry disease, and Tay-Sachs disease. In particular, this screening panel gene was adopted by ACMG (Tier 3) and recommended by Vietnamese experts for the hospitals that conduct carrier screening tests. Those genes were divided into 2 groups based on the detrimental effects on the human body, metabolism disorders and genetic anaemia.

### Next-generation sequencing and bioinformatics data processing

DNA was extracted from peripheral venous blood samples using the QIAamp DNA Blood Micro Kit (Qiagen).

#### DNA extraction

Genomic DNA samples were extracted from peripheral venous blood samples collected in EDTA-coated tubes using QIAamp DSP DNA Blood mini kit (Quiagen, Germany) following the manufacturer’s recommendations.

#### Polymerase chain reaction (PCR)

The thermocycle programme consisted of an initial denaturation at 98 °C for 45s, followed.

by 35 cycles at 98 °C for 15 s, 60 °C for 30 s and 72 °C for 30 s, with a final extension at 72 °C for 1 min. The size and quantity of PCR products were verified by electrophoresis in 2% agarose gel.

#### DNA sequencing

Sequencing on the NextSeq Next-Generation Sequencing System (Illumina, USA): Libraries with a concentration above 10 nM are denatured and sequenced using the NextSeq 500/550 High Output kit on the NextSeq 550 system.

Analysis of Sequencing Results: Sequencing data is backed up and uploaded to a server for analysis. The next-generation sequencing results consist of 75-nucleotide-long DNA sequence reads. Each DNA sequence is aligned to the human reference genome from the National Center for Biotechnology Information (NCBI) using reference GRCh37 to identify its location in the human genome. The position of each sequence read enables the identification of variations occurring in the target gene region. Subsequently, the obtained variants are cross-referenced with databases such as NCBI, Varsome, and OMIM to conclude their characteristics and potential pathogenicity.

### Variant interpretation

In this study, the utilized test is limited to surveying disease-causing mutations in the groups of point mutations, deletions and short insertions (less than 20 nucleotides) in the coding region and the intron neighbourhood (− 20 /+10 nucleotides from each exon) of the surveyed genes; investigation of SEA, − 3.7, − 4.4 mutations for Alpha Thalassemia; no mutations outside the coding region are examined, short continuous repeats, GC-rich regions, regions with high sequence similarity, mitochondrial genes and mosaicism.

### Statistics

Statistical and descriptive analysis was applied using Stata 16.0. These analyses included quantitative variables, such as mean values, standard deviation, minimum and maximum values, and frequency and percentage. The calculation of the rate of pregnant women carrying mutations of recessive genetic diseases in the total number of pregnant women participating in this study.

### Ethics approval and consent to participate

This research involves human participants. Ethical documents were approved by the ethics committee of the GENLAB and Hanoi Medical University (Hanoi, Vietnam) committees. The method abided by the guidelines and adhered to the Declaration of Helsinki. All participants were provided informed consent during the study. All personal information data was encrypted. This study was approved by the ethical committees of the Institute of DNA Technology and Genetic Analysis, Vietnam.

## Results

**Fig. 1:**
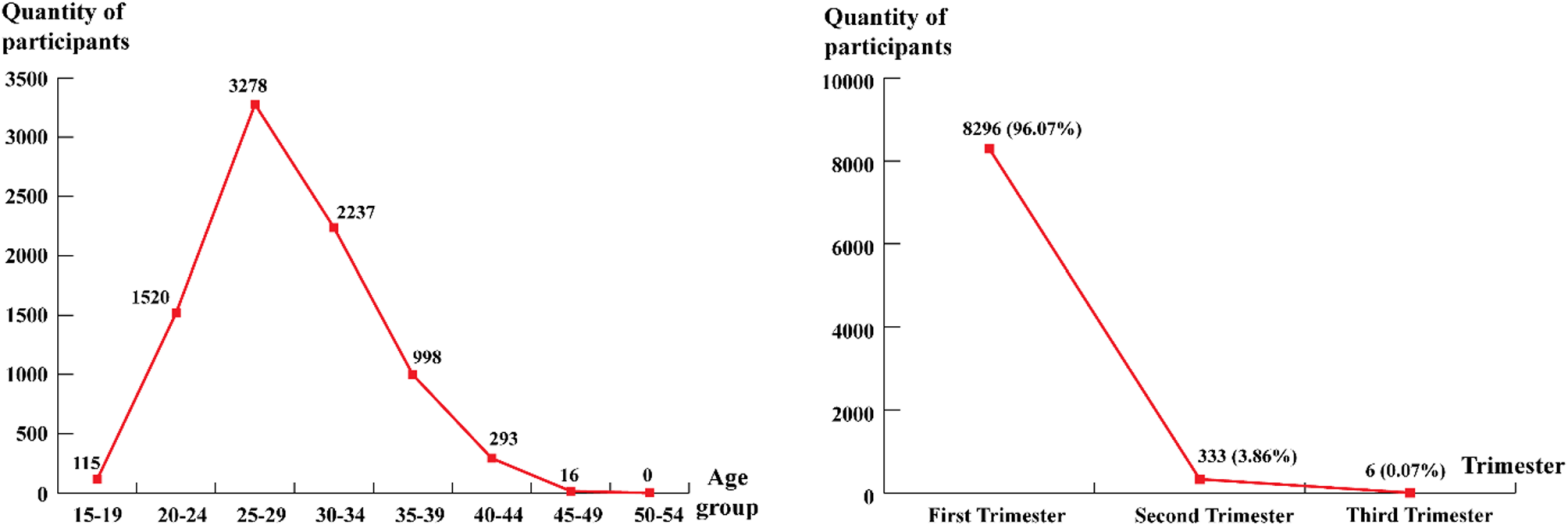
Demographic information.

8.464 Vietnamese pregnant women who had screening tests for 13 recessive conditions at GENLAB from November 1st, 2022, to August 31st, 2023. The mean age of participants was 29,1 ± 5,19 years, and most of them (65,16%) were pregnant with age at 25–35 years. Most pregnant women (96.07%) had this test in the first trimester, only 0.07% had it in the third trimester, and the detailed e frequencies were presented in Fig. 1.

**Fig. 2:**
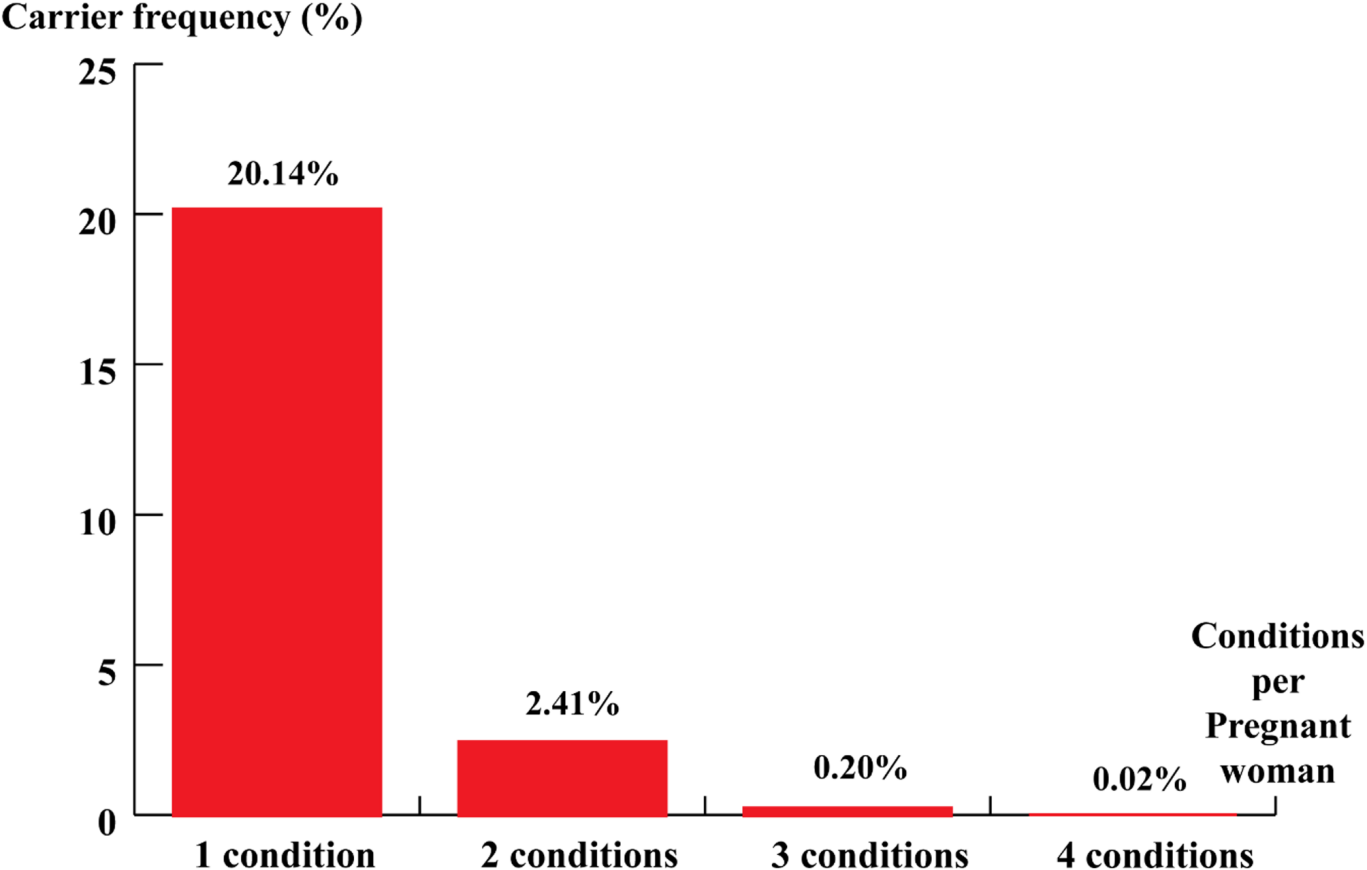
Carrier frequencies of one or more autosomal recessive and X-linked conditions in a pregnant woman.

The overall carrier prevalence for at least one recessive disease was 22.8% and detailed carrier frequencies of recessive conditions were presented in Fig. 2; Table 1. Among 8464 pregnant women tested, 1705 of them (20.14%) carried at least one recessive condition gene, 204 pregnant women (2.41%) were identified as carrying two different recessive condition genes, 17 pregnant women (0.2%) carried three different recessive condition genes, and only 2 pregnant women (0.02%) carried four different recessive condition genes.

**Table 1:** Number and frequency of gene carriers of 13 autosomal recessive and X-linked conditions.

No.	Disease	Gene tested	Number of carriers	Frequency (%)
1	G6PD deficiency	*G6PD*	447	5.28
2	Alpha Thalassemia	*HBA1 & HBA2*	341	4.03
3	5-alpha reductase deficiency	*SRD5A2*	315	3.72
4	Beta Thalassemia	*HBB*	309	3.65
5	Wilson disease	*ATP7B*	215	2.54
6	Phenylketonuria	*PAH*	205	2.42
7	Citrin deficiency	*SLC25A13*	194	2.29
8	Pompe disease	*GAA*	78	0.92
9	Glutaric acidemia type II	*ETFDH*	52	0.61
10	Fabry disease	*GLA*	13	0.15
11	Tay-Sachs disease	*HEXA*	3	0.04
12	Classic galactosemia	*GALT*	0	0
13	Cystic fibrosis	*CFTR*	0	0
Total			2.172	

The most common recessive condition were G6PD enzyme deficiency (carrier frequency 5.28%), alpha thalassemia (carrier frequency 4.03%), 5 alpha-reductase type 2 deficiency (carrier frequency 3.72%) and beta-thalassemia (carrier frequency 3.65%). Conversely, infrequent recessive conditions were Tay-Sachs disease (0.04% carrier frequency), galactosemia (0% carrier frequency) and cystic fibrosis (0% carrier frequency).

Table 2 highlighted the four most prevalent gene mutations for each of the seven most common recessive conditions: G6PD deficiency, Alpha Thalassemia, 5-alpha reductase deficiency, Beta Thalassemia, Wilson disease, Phenylketonuria, and Citrin deficiency. For G6PD deficiency, the c.961G > A (p.Val321Met) and c.1478G > A (p.Arg493His) variants were the most frequent, representing 26.81% and 20.66% of cases, respectively. Alpha Thalassemia predominantly occurs in the form of deletions, with the SEA mutation being the most common (55.97%). In 5-alpha reductase deficiency, the c.680G > A mutation is present in 94.67% of individuals. For Beta Thalassemia, the c.79G > A mutation in exon 1 of the beta-globin chain hads the highest occurrence (65.39%). Wilson disease was characterized primarily by the c.2755 C > G (16.13%) and c.2549 C > T (13.83%) mutations. Lastly, the c.516G > T (p.Gln172His) variant was the most prevalent in Phenylketonuria (65.07%), while c.852_855del (p.Met285fs) dominated in Citrin deficiency, with a frequency of 92.31%.

**Table 2:** Characteristics of several 7 most common recessive carrier conditions gene mutations in the study population.

No.	Mutation type	Changes in protein	Genotype		Number of alleles	Percentage (%)	Frequency (%)
			Homozygous	Heterozygous			
G6PD deficiency (*G6PD* gene)							
1	*c.961G > A*	*p.Val321Met*	3	119	125	26.81	0.738
2	*c.1478G > A*	*p.Arg493His*	0	94	94	20.66	0.555
3	*c.1466G > T*	*p.Arg489Leu*	0	56	56	12.31	0.331
4	*c.1360 C > T*	*p.Arg454Cys*	0	52	52	11.43	0.307
Alpha Thalassemia (*HBA1 & HBA2* gene)							
1	*SEA*		0	197	197	55.97	1.164
2	*-3.7*		0	43	43	12.21	0.254
Beta Thalassemia (*HBB* gene)							
1	*c.79G > A* *(Exon 1)*	*p.Glu27Lys*	2	200	204	65.39	1.205
2	*c.126_129del* *(Exon 2)*	*p.Phe42fs*	0	38	38	12.18	0.225
5-alpha reductase deficiency (*SRD5A2* gene)							
1	*c.680G > A*	*p.Arg227Gln*	5	292	302	94.67	1.784
Wilson disease (*ATP7B* gene)							
1	*c.2755 C > G*	*p.Arg919Gly*	0	35	35	16.13	0.207
2	*c.2549 C > T*	*p.Thr850Ile*	0	30	30	13.83	0.177
Phenylketonuria (*PAH* gene)							
1	*c.516G > T*	*p.Gln172His*	0	136	136	65.07	0.803
Citrin deficiency (*SLC25A13* gene)							
1	*c.852_855del*	*p.Met285fs*	0	180	180	92.31	1.063

## Discussion

### The prevalence of carriers for at least one recessive condition gene

**Fig. 3:**
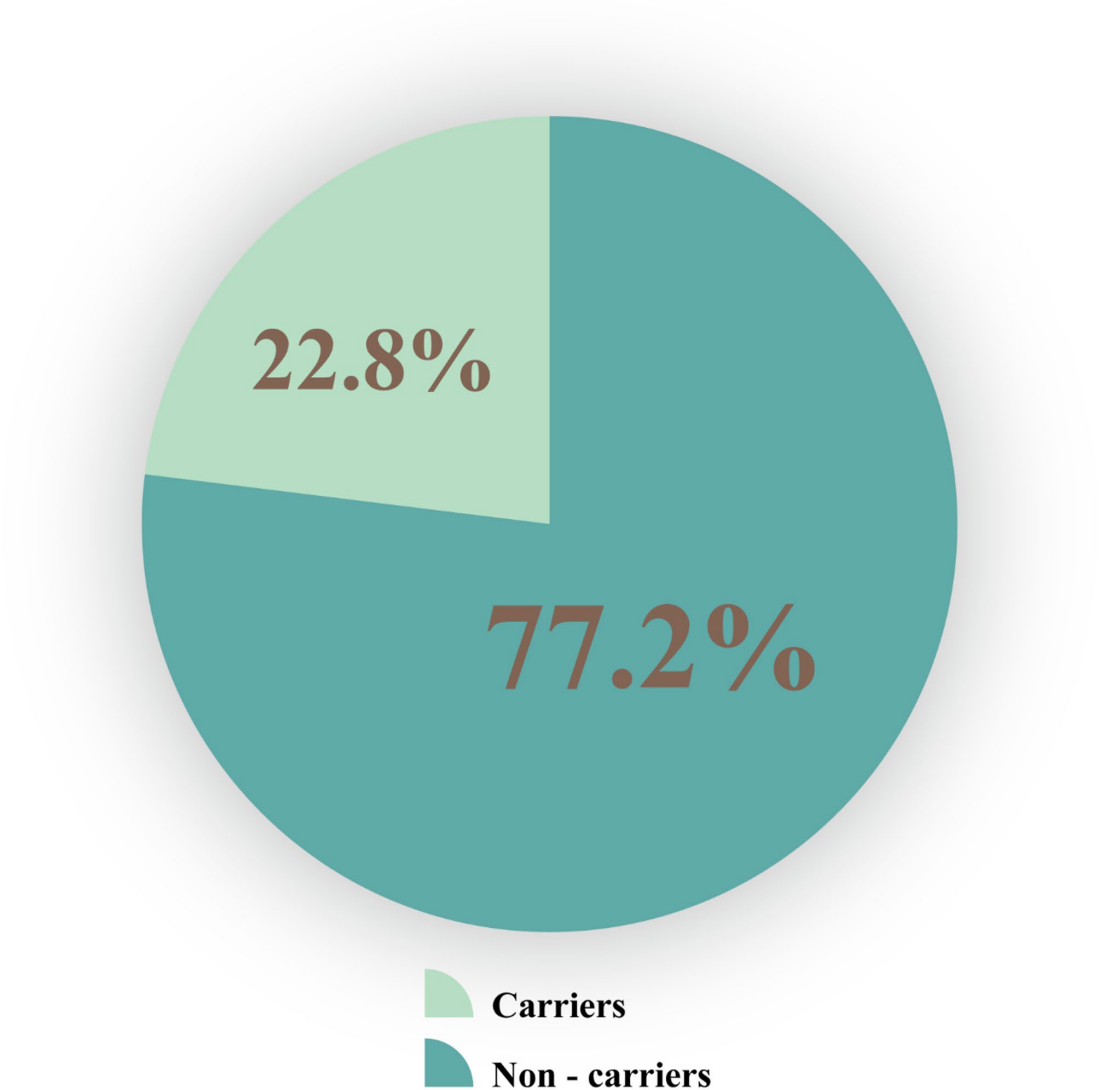
The overall prevalence of carriers for at least one recessive disease.

The study indicated a prevalence of recessive disease carriers at 22.8% among 8,464 pregnant women (Fig. 3). This result was lower compared to a study conducted among women in Southern Vietnam, which reported a carrier rate of 63.6%^13^. The difference in frequency was caused by the utilized carrier screening tests panel, with 564 recessive disorder genes in Southern Vietnam, while only 13 recessive genes were screened in this study. However, when compared to another study conducted in China, Chan et al. implemented an expanded carrier screening program for 104 autosomal and X-linked recessive genetic disorders in 123 pregnant women and their 20 partners, revealing a carrier rate of at least one disorder gene in 58.7% (*n* = 84)^14^. Recently, Chetruengchai et al. analyzed 114 recessive genes in 1642 participants of a carrier screening program in Thailand and determined the carrier frequency of at least one recessive genetic disorder to be 39% (640/1642)^15^. These discrepancies can be attributed to differences in population demographics, sample size, and gene panels employed in carrier screening programs across different regions.

The recessive condition carrier prevalence at 22.8% means at least 1 in 5 pregnant women carried recessive condition genes. Therefore, screening of gene carriers for Vietnamese pregnant women is essential to provide genetic information and prenatal genetic counselling to have appropriate monitoring plans and limit unwanted genetic risks to the fetuses, reducing the disease burden for families and society. Hence, implementing recessive carrier screening tests for pregnant women with affordable costs and effective ways have to be spread across the country and cover all pregnant women.

### The frequency of one or more autosomal recessive and X-linked carrier conditions in a pregnant woman

This study revealed that 20.14% of individuals carried one recessive genetic disease gene, 2.41% carried two distinct recessive disease genes, and 0.2% carried three. These findings indicated that the likelihood of carrying multiple recessive genetic mutations decreases as the number of mutations increases. Additionally, the prevalence of individuals carrying four distinct recessive disease genes was estimated at just 0.02%, or 1 in every 5,000 participants. These results are consistent with a study by Xuan Hong To Mai et al., conducted among women in Southern Vietnam, which also observed a similar trend: as the number of genetic variants in pregnant women increases, their frequency decreases^12^. A comparable tendency was noted in Olivia YM Chan’s expanded carrier screening study, which assessed 104 autosomal and X-linked recessive genetic disorders in 123 pregnant women and 20 of their partners, and in Chetruengchai’s study of 1,642 participants in a carrier screening program in Thailand, which analysed 114 recessive genes^15,16^. Although the exact frequencies vary due to differences in sample size and epidemiological factors, these studies consistently reported that a higher percentage of pregnant women carry multiple genetic conditions compared to those with fewer conditions.

Despite the lower frequency, individuals with a higher number of disease genes have a greater risk of transmitting these genes to their children. This can lead to severe consequences such as the combination of multiple recessive genetic diseases, which, if not detected and diagnosed early, can result in a significant deterioration in the patient’s quality of life and increase the financial burden on the family due to medical treatment. Therefore, carrier screening is essential to provide genetic information and prenatal genetic counselling, allowing for appropriate monitoring plans and reducing the risk of unwanted genetic transmission to the fetus, thereby mitigating the disease burden on families and society.

### Genetic anemia (alpha thalassemia + beta thalassemia + G6PD)

This study showed that pregnant women carrying the G6PD gene variants accounted for 5.28%. This result is higher than some previous reports in Vietnam. To Mai Xuan Hong et al. concluded that 3.57% of women carried the G6DP variants, which is lower than this study^13^. In 2021, a study of five types of diseases at Ho Chi Minh City University of Medicine and Pharmacy found that the prevalence of G6PD deficiency was 3.09%, with men accounting for 68.8% and women for 32.2%^16^. Meanwhile, a study published in 2022 showed that the prevalence of the G6PD gene among Vietnamese pregnant women was 8.9%, which is lower than this study. There was a plausible explanation that the carrier rate varies depending on geographic region and ethnicity^17^. Therefore, it is essential to conduct expanded screening programs for recessive genetic disease carriers to provide recommendations, make additions, and implement mandatory screening programs for high-risk populations in the future. In this study, 19 G6PD variants associated with G6PD deficiency were identified by using next-generation sequencing. The most common variants among Vietnamese ethnic groups were G6PD Viangchan, Canton, Kaiping, and Union, accounting for 71.08% of cases. Additional information on ethnicity, region, and G6PD enzyme activity is needed to assess the correlation between variants and G6PD deficiency based on ethnicity and geography. Due to pregnant women with a homozygous recessive genotype, 100% of male fetuses will inherit the disease gene from their mother and manifest the disease after birth. This means alerting pregnant women and their families to gain more knowledge about the G6PD deficiency disease to provide early screening for the G6PD gene for pregnant women and fetuses to reduce this disease burden in Vietnam. Furthermore, early diagnosis and avoidance of exposure to exogenous oxidative agents are the most effective measures for patients, especially affected children who have significantly higher mortality rates and reduced life expectancy.

Alpha Thalassemia and Beta Thalassemia genes disrupt globin chain synthesis and lead to abnormal haemoglobin production. This condition is a primary cause of hydrops fetalis, responsible for 60–90% of all cases in Southeast Asia^18,19^. In this study, alpha thalassemia and beta thalassemia were the second most common autosomal recessive conditions (4.03% and 3.65%, respectively). Compared to a previous study conducted at the Vietnam Central Obstetrics Hospital in 2021, which found a carrier rate of 12.7% among pregnant women, and a carrier rate of 9.1% in China, the current study showed a lower carrier rate^20,21^. This study identified the most common alpha-thalassemia mutation was the SEA mutation, with a frequency of 1.164% in Vietnamese pregnant women, which was lower than previously reported^22^. Moreover, our findings align with previous studies, which identified − 3.7 mutation as the second most prevalent in Vietnam^23,24^. Among the beta-thalassemia mutations identified, HbE (β:c.79G > A) mutation and c.126_129del (p.Phe42fs) mutation were most frequent. To be specific, HbE, with a prevalence of 65.39%, is the predominant hemoglobinopathy, aligning with studies in Southeast Asia such as Gebhard Flatz et al. reported carrier rates of around 30% in Northeast Thailand and 35% in Laos^25,26^. Furthermore, the c.126_129del mutation on exon 2 was the second most common, accounting for 12.18% of cases. A study by Antonio Cao et al. demonstrated that some early carrier screening programs, molecular diagnosis, and prenatal diagnosis applied to screen for α-thalassemia carriers (the most severe form of α-thalassemia) or β-thalassemia have prevented the development of severe thalassemia in prevalent areas^27^. Moreover, the average lifetime treatment cost for a severe thalassemia patient in Vietnam is a staggering 3 billion VND. Annually, the country requires over 2,000 billion VND to provide essential treatments like blood transfusions and iron chelation for all patients. This significant financial burden, coupled with the need for 500,000 units of safe blood (one-third of the national blood supply), highlights the substantial healthcare demands posed by thalassemia. Therefore, early carrier screening is crucial, not only reducing the disease burden but also alleviating the economic burden and improving the population’s quality of life.

### Metabolism disorders

In this study, the four most common recessive genes of metabolism disorders were identified (> 1/60), including the SRD5A2 gene (5-alpha reductase deficiency), ATP7B gene (Wilson disease), PAH gene (Phenylketonuria), and SLC25A13 gene (Citrin deficiency).

Our findings revealed a higher prevalence of SRD5A2 gene mutation among Vietnamese pregnant women, with 3.72% (315/8464) carrying these mutations. This is notably higher than the 2.3% reported in a previous study, by Tran Ngoc Hieu et al., using a panel of autosomal recessive genes screening^12^. Among the identified SRD5A2 mutations in our study, the c.680G > A (p.Arg227Gln) mutation was the most common, constituting 94.67% of all alleles. This specific mutation results in a substitution of arginine with glutamine at amino acid position 227, which significantly impairs the function of the steroid 5-alpha-reductase 2 enzyme^28^. Due to its rarity, epidemiological data on 5-alpha reductase deficiency is inadequate worldwide. Numerous patients have been raised as girls from infancy until diagnosis, which is often delayed, and face significant health burdens, including severe psychological distress. Furthermore, diagnostic challenges, including complex and time-consuming hormone and genetic testing, limit the accuracy of diagnosis in 20–40% of cases^29,30^. Therefore, the significant prevalence of SRD5A2 gene mutations in Vietnamese pregnant women highlights the importance of carrier screening for autosomal recessive genetic disorders. This screening can help to prevent the birth of affected children and alleviate the associated emotional and financial burdens.

Moreover, this study showed that the frequency of the ATP7B gene (Wilson disease) was high, accounting for 2.54% (or 1 in 40). However, a study in Ho Chi Minh City, Vietnam revealed that the higher prevalence of the ATP7B gene is 1 in 31 (3.3%)^13^. Especially, the most common ATP7B mutation was c.2755 C > G (p.Arg919Gly), accounting for 16,13%, this mutation has been not seen as one of the common mutations in previous studies. According to To-Mai, et al., c.2549 C > T (p.Thr850Ile) was the most common mutation of the ATP7B gene^13^. While the c.3207 C > A (p.His1069Gln) mutation is a common variant in European populations, accounting for 30–72% of cases, it was not identified in any of the Vietnamese individuals examined in this study^31^. Patients with Wilson disease can have severe symptoms, such as liver, neurological, and psychiatric manifestations, and one of them can have neurological complications that include seizures, rigidity, and involuntary movements. Psychiatric disturbances such as personality changes, insomnia, and depression are also commonly observed^32^. To sum up, with the high prevalence of the ATP7B gene among Vietnamese women, prenatal screening and diagnosis are essential to reduce the prevalence of Wilson disease in Vietnam.

The prevalence of PAH mutations was revealed at 2.42%, which is a high carrier gene rate among Vietnamese pregnant women (approximately 1 in 40). Similarly, the carrier rate of 2.5% among 985 individuals was observed in the previous study by Tran Ngoc Hieu et al. (2.5% among 985 individuals)^12^. Nevertheless, a higher carrier rate of PAH mutations was reported at 4.66% by Nguyen Tat Thanh et al. in a larger cohort of 3259 Vietnamese women^33^. Therefore, these varying results highlight the importance of conducting larger-scale studies to accurately determine the prevalence of genetic carriers within the Vietnamese population, especially women because of reproductive health. Moreover, the most common mutation of the PAH gene was identified c.516G > T (p.Gln172His) mutation, accounting for 65.07% among PAH mutations. In South Korea, the c.728G > A (p.Arg243Gln), c.442-1G > A, and Ex6-96 A > G mutations were most common, while the c.842 C > T (p.Pro281Leu) mutation was the most frequent in Iran, accounting for 10.25% of cases^34,35^. Hence, carrier screening for recessive genetic conditions is crucial for identifying couples at risk and providing them with the necessary genetic counselling. Early intervention and management of phenylalanine levels through dietary modifications and supplemental therapies can lead to excellent developmental outcomes for most children with PKU by the age of five.

Due to the importance of the pathway of glucose metabolism and the urea cycle in the human body, reducing the prevalence of Citrin deficiency is vital to ensure lifelong health and human expectancy. However, this study showed that the prevalence of SLC25A13 gene mutations was 2.29%, which means at least one pregnant woman carries SLC25A13 gene mutations among 45 pregnant women. This result is lower than the previous study in Vietnam, with 3.23% (1 in 31) by Tran Ngoc Hieu et al., but it was still a higher prevalence and was required to be screened before couples wanted to have any child^12^. The most common SLC25A13 mutation was identified c.852_855del (p.Met285fs), accounting for 92.31%. Similarly, the most common mutation of SLC25A13 was c.852_855del (p.Met285fs) by Tran Ngoc Hieu et al., which is consistent with findings from other populations such as China and Japan, due to this mutation, it has been established as a major hotspot for citrin deficiency^36–38^.

While this study provides valuable insights into the genetic landscape of autosomal recessive and X-linked conditions among Vietnamese pregnant women, several limitations should be considered. First, the non-randomized selection of participants may introduce selection bias, and the sample’s limited geographic and ethnic diversity—primarily from urban and semi-urban areas of Northern Vietnam with a predominance of Vietnamese Kinh ethnicity—may not fully represent the broader Vietnamese population. Second, the study focused exclusively on 13 selected recessive conditions, potentially excluding other relevant genetic disorders. Additionally, two specific genes, GALT and CFTR, were not identified in this study. This absence could be attributed to factors such as the limited sample size, the specific genetic makeup of the population studied, or the rarity of these variants. Further research with larger and more diverse cohorts is needed to fully understand the prevalence of these genes. Moreover, due to resource constraints, Sanger sequencing was not conducted to confirm the identified mutations. Instead, variant identification relied on stringent bioinformatics pipelines and cross-referencing with established databases. This approach, while robust, may have limitations in detecting certain variants, particularly novel or rare mutations not well-documented in existing databases.

## Conclusion

The prevalence of recessive carrier genes among Vietnamese pregnant women is high. Promoting and raising awareness of recessive carrier screening tests is essential to guide and aid reproductive health counselling for individuals and their families. This is the first study to analyse the largest result database of the commonly utilized test about some recessive carrier condition genes in Vietnam to describe the prevalence of these carrier genes, which leads to a better understanding of the Vietnamese recessive genetic landscape. However, the study’s findings are drawn from a non-randomized selection of participants, predominantly from urban and semi-urban areas of Northern Vietnam and largely of Vietnamese Kinh ethnicity. As a result, the generalizability of these findings to the broader Vietnamese population may be limited, given potential genetic variations across different regions and ethnic groups. Nevertheless, this research provides a foundational step toward a deeper understanding of recessive genetic traits in Vietnam, paving the way for future studies with more diverse and representative samples.

## Data Availability

While the data used in this study comes from the Institute of DNA Technology and Genetic Analysis, there are limitations on its public availability. We obtained the data under a specific license for this research and cannot share it publicly. Data was available online: 10.6084/m9.figshare.28078850.
